# Brain Stimulation in Neurology and Psychiatry

**DOI:** 10.1111/j.1749-6632.2012.06650.x

**Published:** 2012-07-25

**Authors:** Simon Little, Peter Brown

**Affiliations:** Nuffield Departments of Clinical Neurosciences, University of OxfordOxford, United Kingdom

**Keywords:** Parkinson's, DBS, feedback control, LFP, beta

## Abstract

Feedback control of deep brain stimulation (DBS) in Parkinson's disease has great potential to improve efficacy, reduce side effects, and decrease the cost of treatment. In this, the timing and intensity of stimulation are titrated according to biomarkers that capture current clinical state. Stimulation may be at standard high frequency or intelligently patterned to directly modify specific pathological rhythms. The search for and validation of appropriate feedback signals are therefore crucial. Signals recorded from the DBS electrode currently appear to be the most promising source of feedback. In particular, beta-frequency band oscillations in the local field potential recorded at the stimulation target may capture variation in bradykinesia and rigidity across patients, but this remains to be confirmed *within* patients. Biomarkers that reliably reflect other impairments, such as tremor, also need to be established. Finally, whether brain signals are causally important needs to be established before stimulation can be specifically patterned rather than delivered at empirically defined high frequency.

## Introduction

Deep brain stimulation (DBS) has been in routine clinical use for more than a decade and provides a highly valuable treatment modality for selected patients with Parkinson's disease (PD) in the management of uncontrolled motor symptoms. DBS has been shown to significantly improve motor control, reduce requirement for the dopamine prodrug, levodopa, and improve quality of life over best medical treatment.^1^

Nevertheless, the cost of functional neurosurgery, its partial efficacy, and its side effects mean that there is still a pressing need for improvement. With DBS as currently used, stimulation is always on, relentlessly interfering with neural circuits regardless of the level of pathological activity. This, therefore, shortens battery life and may promote habituation and side effects such as impairment of verbal fluency, neuropsychiatric symptoms, and paradoxical worsening of motor functioning.^2^ One way to improve DBS is to deliver stimulation in a closed-loop mode. With this approach, stimulation is delivered according to clinical state so that networks are stimulated only when necessary, saving on battery power, limiting habituation, and improving patient outcome. Closed-loop stimulation has already proved very effective in the treatment of cardiac arrhythmias and is being trialed in epilepsy.^3–6^

Another way that DBS could be improved is to optimize the precise pattern of stimulation delivered. Standard DBS for PD is usually delivered at 130 Hz with a power or 1–4 V and pulse width of 60 μs. These parameters have been selected on empirical grounds but the available parameter space has not been fully searched and the possibility of strategically patterning stimulation according to the form and phase of pathological network activity has been barely explored. Yet patterned stimulation regimes that specifically target pathological activities may potentially be more efficient and have less effect on physiological processing, and thereby have less power consumption and fewer side effects.

Both these approaches to improving DBS require a detailed understanding of pathological brain signals. In closed-loop DBS, when and where to stimulate is determined by clinical state, there is no kinematic measure that can comprehensively capture diverse impairments such as rigidity, slowness of movement, or tremor, and yet be miniaturized. Hence, the search is on for brain signals that faithfully reflect clinical state and can be measured with minimal extra intervention. Such a signal should be sensitive and specific to the clinical state and reliable over time and in different conditions. Yet it should also provide a more-or-less instantaneous measure of clinical state so that therapy does not lag impairment. Moreover, it should be calculable with the lowest possible computational requirement as this will affect both battery consumption and time to change in the efferent limb of any control system. In the case of strategically patterned stimulation the requirements are even more stringent, for the brain signal must not merely correlate with clinical state but must also cause it in order to be a legitimate target. Unsurprisingly, there is as yet no single biomarker that fulfills all of these criteria.

Parkinson's disease is classically defined as the triad of tremor, rigidity, and bradykinesia. When considering feedback parameters for PD, therefore, one must first establish whether the different aspects of the disease represent different manifestations of a single pathological system that could be potentially represented by a single biomarker or whether these different motor manifestations have different substrates and are therefore likely to be represented by separate biomarkers. There is now strong evidence for more than one system and specifically it appears that rigidity–bradykinesia is functionally discrete from tremor.^7^ This dichotomy is further complicated when one considers other well-recognized PD symptoms such as gait disorders and nonmotor symptoms. Thus, the dominant phenotype of the patient should direct the choice of biomarker or biomarkers for closed-loop DBS in an individual patient. Fortunately, growing evidence that disturbed temporal coding at the neuronal and population level may lie at the heart of Parkinsonian impairment affords hope that different biomarkers may be organized in the frequency domain and available through recordings from the same site.^8^

## Cortical signals

Given the location of the DBS electrodes precisely at the site of interest, namely within the pathological basal ganglia circuit itself, it seems most rational to seek potential biomarkers within this region, and, preferably, to record these through the stimulating electrode itself. On the other hand, an advantage of recording relevant biomarkers away from the stimulation site would be avoidance of contamination via stimulation artifact, opening up the possibility of using biomarkers over a wider frequency band, as current approaches to recording from stimulation sites rely on low-pass filtering to recover biological signal.^9,10^ In light of this, it is worth first considering the possibility of deriving suitable biomarkers from the cerebral cortex. The electroencephalogram (EEG) has been the predominant signal used for brain computer interfacing (BCI) previously, due to its noninvasive nature. It records the aggregate signal from a surface cortical population of an area of about 6 cm^2^ and has successfully been used to control simple movements in healthy volunteers and in patients.^11^ EEG signals in patients even with early untreated PD have been shown to be distinguishable from age-matched controls by both linear and nonlinear analyses, but only at the group level.^12,13^ So far only a handful of studies have demonstrated a correlation between EEG-derived measures and motor impairment, and between treatment-induced change in such measures and changes in motor impairment.^14,15^ These EEG-based measures are based on interregional synchronization and need extensive head coverage so that EEG and electrocorticography (ECoG) are currently impractical approaches to long-term closed-loop control. Moreover, it remains to be shown that correlations with motor impairment can be made within subjects as state changes over time, and not just across subjects.

An alternative approach to deriving cortical signals is to record the activity of single or multiple units in the cortex. This affords remarkable spatial resolution, but is potentially at the cost of the loss of diffuse population-coded information. The latter is often addressed by the use of multiple microelectrodes. However, there are considerable technical limitations to their application in the chronic setting that has thus far limited their widespread clinical use. It has been found that only approximately one half of implanted microwires deliver recordable units and this further deteriorates over time due to neuronal gliosis around the electrode tips and other hardware failures.^16^ Additionally, there is a significant computational cost involved in the online analysis of multiple discrete electrode signals of this sort.

## Basal ganglia signals

Most of the attention has been focused on recording potential biomarkers directly from the basal ganglia in PD. Again, there are several approaches. One is to record single units and Weinberger *et al.* showed not only that there was increased burst activity on recording from individual neurons in the off-drug state but that there was also a significant correlation between the incidence of oscillatory neurons and the patient's benefit from dopaminergic medications, although this correlation did not extend to motor deficit at rest, off medications.^17^ A related approach is to record the background activity from the microelectrode. Individual neuronal spikes are selectively removed from the data and replaced with randomly selected surrogate spike-free data from the same trace. The data are then high-pass filtered at 70 Hz to remove synaptic potentials. Signals processed in this way are thought to represent the highly localized aggregate activity of action potentials of the population of neurons very close to the microelectrode tip.^18^ This too has been shown to correlate with motor impairment but its utility in closed-loop stimulation regimes may be limited by the need to reject from the signal both unit activity and stimulation artefact, which share a similar frequency content.

Intracranial microdialysis provides a very different approach and is already routinely used in the research setting in animal models of Parkinson's and in humans for other conditions such as posttraumatic head injury for real-time and continuous monitoring of cerebral metabolites.^19^ It is possible that dopamine or dopamine metabolites from the striatum may provide a useful biomarker of clinical state that could be used for closed-loop stimulation. Some newer silicon electrodes have microdialysis tubules included within them and show that microdialysis can be integrated into stimulation electrodes.^16^ However, there may be significant technical barriers to implementing this type of approach including tube blockage and miniaturization of the chemical analysis hardware for implementation in an implantable device. Moreover, it seems unlikely that microdialysis will have the temporal resolution to avoid lags between clinical change and therapy titration. More suitable may be fast scan voltammetry, which is an electroanalytical technique that extracts information on a subsecond temporal scale regarding the chemical composition of the extracellular fluid by varying the potential at a microelectrode and measuring the evoked current. This has successfully been piloted in a large animal model to show that DBS elicits a time-locked release of dopamine that is both intensity and frequency dependent.^20^

## Local field potentials

Despite these endeavors, most research has concentrated on the use of the LFP that can be recorded from the contacts at the end of the very same electrode used for chronic stimulation and so requires no additional electrodes or hardware. Typically the LFP is picked up from the subthalamic nucleus (STN), where, although highly focal and localized, it still represents a population averaged signal. It is recorded at lower frequencies than single-unit recordings, which is believed to be beneficial since these lower frequencies are less affected by electrode interface or by local geometry.^16^ Reassuringly, research has still shown that the LFP potentials are closely related to the activity of individual neurons with synchronized bursting of neurons occurring in phase with beta (13–30 Hz) activity off medication.^17,21^ In light of the averaged nature of LFP activity across a population of neurons, it could be questioned whether these signals are appropriate for monitoring of complex state dynamics. However, it should be noted that within the basal ganglia and particularly the STN, there is great convergence of information processing from across the cortex into the localized area around the LFP recording site. Additionally, a population-based metric may well be superior to that of single unit recordings, given that many states are represented diffusely across populations rather than within single neurons, and this is likely to be particularly the case when tracking general state changes in PD rather than the subtle and highly localized motor coding that is involved in precise voluntary movements. Indeed, in some situations, LFPs are superior to single units for representing movements.^22^ These considerations, combined with the long-term stability of DBS at the tissue-electrode interface, make LFPs very attractive feedback control parameters for responsive DBS. But how informative are they about different clinical features?

### Bradykinesia and rigidity

**Figure 1:**
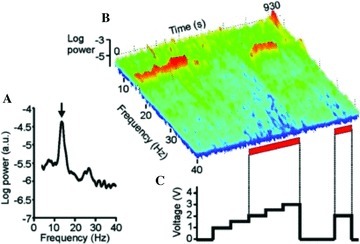
Effect of deep brain stimulation (DBS) of subthalamic nucleus on the local field potential (LFP). (A) Power autospectrum of LFP recorded without stimulation. There is a large peak arrowed at 14 Hz. (B) Frequency–time log power spectrum of LFP. Power, as in (A), shown over the pass band of the amplifier (4–40 Hz). Red bars along the time axis denote periods of DBS at 2.0–3.0 V. Dyskinesias of the contralateral foot were noted at voltages of 2.0 V and above. Note suppression of spectral peak with stimulation ≥2.0 V, with evidence of a temporary increase in the power of the peak with stimulation at 1.5 V and a delayed return of the peak after stimulation at 3.0 V is terminated. (C) Timing and voltage of DBS. Adapted from Eusebio *et al*.,^10^ with permission.

Early recordings in PD patients with DBS electrodes revealed that when recorded in the off-medication state, power spectra showed high levels of synchronized activity between 13 Hz and 30 Hz (beta-frequency band) in both the STN and the globus pallidus interna (GPi) and subsequent follow-up studies have confirmed this in the majority of cases (Table 1). It has been shown that beta activity is suppressed with levodopa treatment and that the degree of suppression correlates with motor improvement measured by the UPDRS clinical rating scale, particularly in the range of <20 Hz.^23–25^ It has recently been demonstrated that DBS treatment also suppresses beta activity and STN-DBS–driven improvement in rigidity and bradykinesia (but not tremor) correlates with suppression of synchronization (Fig. 1).^10^^,^^26–30^ Reassuringly, it also appears that the beta profile is stable within patients over time and following DBS treatment.^27,28,31^

**Table 1:** Studies that have recorded from the STN with the proportion of patients or nuclei (in brackets) that have demonstrated beta peaks in the off state at rest

Group	Year	No. of patients/(nuclei) recorded	Number with beta peaks patients or (nuclei)	%
Brown^41^	2001	4	4	100
Cassidy *et al.*^34^	2002	6	6	100
Levy *et al.*^35^	2002	14	9	64
Silberstein *et al.*^79^	2003	(17)	(17)	100
Kuhn *et al.*^65^	2004	8	8	100
Priori *et al.*^80^	2004	(20)	(17) low beta	85
Kuhn *et al.*^21^	2005	6 (8)	(8)	100
Doyle *et al.*^81^	2005	14	14	100
Wingeier *et al.*^30^	2006	4 (6)	(6)	100
Foffani *et al.*^28^	2006	(13)	(11) low beta	85
Kuhn *et al.*^23^	2006	9 (18)	(17)	94
Kuhn *et al.*^82^	2006	8	8	100
Alonso-Frech *et al.*^48^	2006	(28)	(28)	100
Weinberger *et al.*^17^	2006	14	14	100
Ray *et al.*^25^	2008	(13)	(11)	85
Bronte-Stewart *et al.*^27^	2009	(22)	(22)	100
Kuhn *et al.*^24^	2009	30 (57)	(52)	89
De Solages *et al.*^63^	2010	(28)	(28)	100
Pogosyan *et al.*^61^	2010	18	18	100
Eusebio *et al.*^10^	2011	16 (28)	(25)	89

The mean proportion of patients/nuclei showing beta peaks is 95%. The whole beta band is considered, except where otherwise indicated. A number of studies have also reported beta activity but not explicitly stated the number of peaks detected.^47,64,69^^,^^81–85^

However, several studies have failed to show a correlation between the Unified Parkinson's Disease rating scale (UPDRS) motor score and beta activity at rest across patients, leading some to downplay its significance.^17,23–25^ Additionally, a minority of patients fail to show a substantial beta peak off medication (Table 1). A number of reasons have been proposed to explain these conflicting results. Some have argued that this may be due to a “stun effect” from postoperative localized edema, which causes a transient improvement in symptoms even without medication or the stimulator being initiated.^32^ Alternative considerations include variability in electrode targeting between patients, the weaknesses of the UPDRS clinical scale, and issues related to signal normalization. A causal role for beta activity in the pathophysiology of PD has also been sought, with experiments showing that stimulation at beta band frequencies generally cause slowing of movement, albeit modest (Table 2). Although there is still debate surrounding beta in PD, in particular its causal role, it clearly appears that in the majority of patients beta is present at rest in the off state (mean 95%, see Table 1) and may therefore be useful as a feedback signal for DBS. Encouragingly, it has been shown that despite the enormous differential in magnitude between recorded beta and DBS stimulation voltage, one can successfully monitor beta during DBS.^9,10^

**Table 2:** Studies that have attempted to demonstrate a causal role for low-frequency oscillations by stimulating at low frequency in PD patients withdrawn from their usual medication

Group	Date	No. of patients	Task (off/on medication)	Effective frequency (Hz)	Effect size
Timmermann *et al.*^83^	2004	7	UPDRS akinesia (off)	10	10% increase motor UPDRS
Fogelson *et al.*^84^	2005	10	Finger tapping (off)	20	6% slowing
Chen *et al.*^85^	2007	22	Finger tapping (off)	20	8% slowing
Eusebio *et al.*^86^	2008	18	Finger tapping (off)	5 and 20	12% slowing
Chen *et al.*^85^	2011	16	Grip force (off)	20	15% slowing of force development
Little *et al.*^87^	2012	12	Rigidity (on)	5, 10, 20	24% increase in rigidity

Thus far, we have examined LFP signals at rest and their relationship to rigidity and bradykinesia. A useful feedback parameter, however, must also reliably indicate state information during more complex situations including voluntary and cued movements. Prior to and during movement, synchronized beta oscillatory activity is reduced; this has been shown at the single-unit, LFP, and cortical levels.^33–37^ Cassidy *et al.* found that not only was there increased beta activity off-medication in the GPi and STN and that these were reduced with levodopa, but that coupling through coherence was also reduced between these two nuclei during action. They also found that in the on state, LFP activity was dominated at higher frequencies in the gamma range (70–85 Hz) and this was augmented with movement, displaying a double dissociation between off and on drug states and changes with action in the beta versus gamma band. Foffani *et al.* have shown that not only does beta amplitude change during movement but the frequency of the prominent peaks also changes slightly in response to movement and dopamine therapy.^38^ This suggests that amplitude modulation and frequency modulation might both be important for coding motor state.

Physiological states other than movement also modulate LFP rhythms, including beta rhythms. It has been shown, for example, that during slow-wave sleep, beta activity is reduced, whereas in rapid eye-movement sleep, beta is similar to—or possibly greater—than in the awake state.^39^ It has also been found that motor control improves during sleep in those with PD.^40^ Thus, monitoring of beta might also signal non-REM sleep, affording the potential for DBS to be turned off, thereby saving on power consumption if implemented in a closed-loop setup.

The changes in LFP signals prior, during, and after movements, and during sleep, show the complex relationship between brain rhythms and the physiological state in patients with PD. If beta, in particular, were to be used as a biomarker for feedback control, one concern given the preemptive suppression prior to movement would be that if stimulation were to be turned off at this critical period in response to beta reduction, then freezing of action or gait may be exacerbated. Consideration to the length of the beta-averaging window may alleviate this problem. Beta therefore appears to be a pathological signal in the PD off state with a relationship to bradykinesia–rigidity. Other studies have suggested that this bradykinetic signal may be balanced by a prokinetic one, namely gamma,^41^ and that the relationship between the two is reciprocal.^42^ Gamma activity is related directly to movement and EEG studies have demonstrated that it is differentially involved in ballistic versus negative movements.^36^ Thus gamma and beta seem to have complimentary roles in motor coding both at the cortical and subcortical levels.

### Tremor

As described previously, it appears that tremor and bradykinesia–rigidity are subserved by different pathophysiological systems and, therefore, may require separate neurophysiological biomarkers to adequately capture them. At the cellular level, Levy *et al.* showed that in PD patients with tremor, there exists a subsection of neurons that discharge coherently with the limb tremor that are themselves characterized by both in and out of phase relationships to the tremor. In addition, they demonstrated high-frequency synchronization during tremor periods and suppression of synchronization during voluntary movement.^35,43^ Analysis at the level of the LFP has demonstrated increased signal at the tremor frequency and also double the tremor frequency (likely representing a harmonic), which was well localized within the STN and coherent with peripheral EMG.^44^ Investigation of activity at the cortex has also shown that this activity is coherent with the M1 motor area and extends out to a wider, diffuse network including other cortical areas, the cerebellum, and diencephalon.^45^ Further work using nonlinear techniques has revealed that the coupling in the theta band is bidirectional between the LFP and the peripheral tremor and there is significant delay (1–2 tremor cycles) for the brain-to-tremor driving signal.^46,47^ In addition to tremor frequency oscillations, the beta band appears functionally related to tremor showing beta suppression prior to the onset of resting tremor.^47^ This effect, which is opposite to the positive correlations already described between beta activity and bradykinesia–rigidity, poses a problem for the use of beta activity as a biomarker in patients with PD, particularly those with tremor, and reinforces the need to individualize biomarkers according to clinical phenotype or to combine biomarkers. Overall, very little work has been done in terms of demonstrating a correlation between single unit or LFP activity and tremor severity over time.

### Dystonia and dyskinesias

Further aspects of Parkinsonism that may be reflected in the LFP are dystonia and dyskinesias. Alonso-Frech *et al.* have investigated LFP signals while inducing dyskinesias in patients with PD through treatment with levodopa or apomorphine.^48^ They found in patients who manifested dyskinesias a large increase in activity in the 4–10 Hz band, and when dyskinesia was present in just one limb, it was found that the 4–10 Hz activity was present on the contralateral side only, suggesting that it is specific to this clinical feature. On the other hand, dystonia and dyskinesias have been inversely related to beta power levels.^49^ This raises the possibility that the suppression of beta may favor the development of hyperkinesias, under some circumstances, and reinforces the view that closed-loop control driven by a single biomarker, such as LFP beta band power, may be insufficiently nuanced to apply to all impairments. In addition, the relative merits of different basal ganglia targets for the sensing of biomarkers sensitive to dystonia and dyskinesia are unclear.

### Gait and nonmotor symptoms

The above characteristics refer to LFP signals in relation to motor symptoms. PD, however, extends to many other impairments including motor features like gait freezing, and nonmotor symptoms such as depression, sleep disturbance, postural instability, and autonomic dysfunction. Is it possible that specific biomarkers exist that may allow dynamic modulation of DBS specific to these impairments as well? There has been little research as of yet into specific physiological biomarkers of the nonmotor symptoms; however, progress is being made into a better understanding of the pathophysiology of gait with specific reference to a new subcortical target, namely the pedunculopontine nucleus (PPN). Interestingly, this target is most effective when stimulated at low frequency and is found to have a specific therapeutic effect on gait and postural instability.^50^ Both beta and alpha activity have been recorded in the PPN, and are at least partially dopamine dependent and coupled to cortical activity. ^51,52^ At present, although there is an insufficient understanding of PPN LFPs to relate individual signal components directly to relevant clinical features, there is emerging evidence that PPN signals may provide dynamic information related to gait.

### Higher-order spectral analyses

The previous examples demonstrate that important and meaningful information regarding aspects of the concurrent pathological state can be derived from simple spectral analysis of the LFP signal. Activity has thus been divided into individual frequency bands that have been related to different aspects of the disease/medication state through the use of correlations. However, this approach makes the assumption of a linear relationship between signal and clinical feature. Although some coding at the population level may occur in a linear manner, this likely does not completely describe how the brain communicates, and it is therefore possible that some valuable information regarding state is not being captured with these simple approaches.^53,54^ More sophisticated analyses have therefore been examined. The notion of “complexity” relates to dynamical systems theory and simply put, describes systems that are somewhere between simply predictable deterministic systems and those that show chaos. Complex systems appear to occur more readily in systems that are far from their natural equilibrium and can be recognized by characteristic features such as power law scaling (1/f), fractals, and self-similarity.^55^ Quantitative measures of complexity have been developed and one such measure, Lempel-Ziv complexity, has been used to show that the complexity of the LFP in the beta-frequency range negatively correlates with bradykinesia–rigidity at rest, but not with tremor.^56^ As synchronized oscillatory activity increases, complexity falls, and the system becomes more deterministic and it is possible that complexity in the beta band directly relates to PD symptoms. Alternatively it may be that this complexity measure simply normalizes the signal and thus avoids some of the experimental confounds highlighted previously (for example, stun effect and electrode targeting). Further recent evidence suggests that variability of beta-band power can inform on clinical state both at rest and in response to dopa.^57^

**Figure 2:**
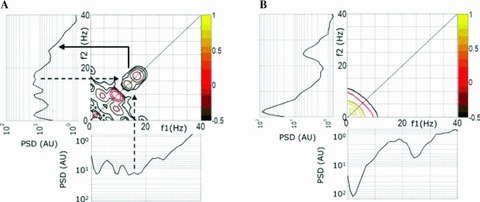
Bispectral analysis. Mean bispectrum from 13 subthalamic nuclei before (A) and after (B) levodopa administration. The central 2-D plot shows the mean bispectrum of the LFP signals as a function of frequencies f1 (*x*-axis, in Hz) and f2 (*y*-axis, in Hz). The level lines in the plot represent bispectrum values color coded as indicated in the color bar on the right (log transform of the average bispectrum, expressed in log arbitrary units, log AU). The mean power spectrum is also shown adjacent to each axis. The diagonal in the central plot defines the two regions of symmetry of the bispectrum. (A) Before levodopa administration, the arrows indicate the harmonic nonlinear correlation between the LFP rhythm in the low-beta range (13–20 Hz, dashed lines) and the LFP rhythm in the high-beta range (20–35 Hz, continuous line). This nonlinear correlation is evidenced by the bispectral peak (13–20 Hz, 13–20 Hz). Note that this bispectral peak appears broad due to the frequency variability between nuclei. Bispectral peaks are also present in other regions; (2–7 Hz, 2–7 Hz), (8–12 Hz, 8–12 Hz), and (2–7 Hz, 8–12 Hz), suggesting the presence of nonlinear correlations between different LFP rhythms in the off Parkinsonian state. (B) After levodopa administration, bispectral peaks are suppressed. The mean spectral peak in the high-beta range is therefore now independent of activity at lower frequencies. Adapted from Marceglia *et al*.,^58^ with permission.

Phase relationships between frequencies within the same signal may also be informative. Marceglia *et al.* have shown using bispectral techniques that LFP signals become nonlinearly correlated in the absence of dopamine and that this is particularly strong between low and high beta (Fig. 2).^58^ The bispectrum relates to phase–phase interactions; however, the introduction of the investigation of phase relationships raises other possibilities. Might phase–amplitude interactions between different frequencies also contain significant information? Indeed, it has recently been shown that the degree of movement-related modulation of high-frequency oscillations by beta negatively correlates with bradykinesia/rigidity scores and that in the on-medication state the high-frequency oscillations are released from lower-frequency coupling and demonstrate marked amplitude modulation related to movement.^59^ Other recent work has examined the role of these high-frequency oscillations themselves rather than their relationship to beta and has shown two distinct high frequency bands centered around 250 Hz and 350 Hz.^60^ Moreover, they found that the power ratio of these two bands correlated with UPDRS bradykinesia–rigidity at rest and that this was dopamine dependent and unrelated to beta. Nonlinear analyses are potentially interesting but their correlation with motor impairment does not so far substantially differ from that seen with simple power measures and yet they come with a computational cost that may limit their application to clinical closed loop DBS in the near future. However, no study so far has directly contrasted correlations between linear and nonlinear measures and clinical impairment, nor determined whether those cases that contribute to correlations are the same for the two approaches. If not, then the two approaches may prove complementary. Table 3 summarizes the many studies that have related electrophysiological features to clinical state and highlights the relative lack of studies looking at correlations *within* subjects.

**Table 3:** Studies that have investigated the relationship between electrophysiological signals and clinical features

Technique/site	Correlation between treatment-induced changes in brain signals and impairment across subjects	Correlation between treatment-induced changes in brain signals and impairment within subjects	Correlation between brain signals and clinical state or change in clinical state across subjects	Correlation between brain signals and clinical state or change in clinical state within subjects
**Cortical**				
EEG-EEG coherence, Silberstein *et al.*^14^	Reduction in EEG-EEG coherence (including beta) correlates with UPDRS improvement (levodopa and DBS); no *r* values given.		Beta band EEG-EEG coherence correlates with UPDRS; no *r* values given.	
MEG synchronization likelihood (SL), Stoffers *et al.*^15^			Positive association of UPDRS with interhemispheric (η^2^= 13.4%) and intrahemispheric (η^2^= 12.3%) theta and interhemispheric beta (η^2^= 9.2%) SL measures	
**Single/multiunit**				
Single unit recordings, Weinberger *et al.*^17^			Negative correlation between percentages of beta oscillatory cells with on drug motor UPDRS (*r*^2^= 0.49) Positive correlation between percentage of beta oscillatory cells with preop levodopa response; (*r*^2^= 0.62)	
Spectral density estimation of multiunit activity, Zaidel *et al.*^88^			Spatial extent of STN beta oscillations positively correlates with improvement on DBS and levodopa (*r*^2^= 0.45) Correlation between beta power off drugs with improvement in motor UPDRS with levodopa *r*^2^= 0.2 or DBS (*r*^2^= 0.3)	
**Local field potentials**				
LFP power spectral densities, Kühn *et al.*^23^	Reduction in beta with levodopa correlates with improvement in contralateral motor UPDRS (ρ= 0.81)			
LFP power spectral densities, Ray *et al.*^25^	Reduction in beta levodopa correlates with improvement in contralateral bradykinesia/ rigidity UPDRS (ρ= 0.7)		Baseline beta power off-medication correlates with improvements in motor symptoms (ρ= 0.68)	
LFP amplitude modulation by movement (cross- correlation index), Androulidakis *et al.*^37^			Tapping performance versus beta cross-correlation index across patients (*r*^2^= 0.14)	Tapping performance versus beta cross correlation index within patients (*r*^2^≤ 0.53)
LFP power spectral densities during consecutive DBS/ LFP recordings Kühn *et al.*^26^	Reduction in beta following DBS correlates with improvement in contralateral bradykinesia. *r*^2^= 0.31			
LFP power spectral densities (frequency aligned), Kühn *et al.*^24^	Reduction in beta with levodopa correlates with improvement in contralateral bradykinesia/rigidity UPDRS together (*r*^2^= 0.38), rigidity alone (*r*^2^= 0.34) and bradykinesia alone (*r*^2^= 0.17)			
LFP beta spatial extent (phase synchrony), Pogosyan *et al.*^61^			Phase coherence across contacts correlated with bradykinesia/rigidity. (*r*^2^= 0.24)	
LFP power spectra—high-frequency oscillations (HFO), López-Azcárate *et al.*^59^			Movement-related modulation of the HFOs negatively correlates with rigidity/bradykinesia scores (*r*^2^= 0.39)	
LFP Lempel-Ziv complexity, Chen *et al.*^56^			Negative correlation of beta band complexity with akinesia–rigidity (ρ=−0.54)	
LFP power spectra—high-frequency oscillations (HFO), Ozkurt *et al.*^60^			Power ratio of 250Hz and 350Hz HFOs correlates with UPDRS akinesia/rigidity (ρ= 0.36)	
Beta variability (coefficient of variation—beta Power), Little *et al.*^57^	Change in CV of high beta negatively correlates with changes in UPDRS in response to Levodopa (ρ=−0.66)		CV of high beta negatively correlates with UPDRS at rest (ρ=−0.59)	

Note: This table demonstrates a clear relationship between LFP activity (particularly beta) and change in clinical features across subjects but highlights the very limited evidence for LFP signal correlations with clinical state within subjects. In the beta-frequency band, the sign of any correlation is always consistent with the fact that high levels of LFP activity are associated with a worse clinical state and larger decreases in lfp activity with treatment are associated with greater improvements in clinical state. Note that improvement is predominantly in bradykinesia-rigidity. Where possible, *r*^2^ and η^2^ values are given so as to show the proportion of the variance in clinical scores that can be predicted by the brain signals.

## Multiple sites

There is emerging evidence that signals at all levels including cortex, LFP, and single units, contain information that represents the clinical state and that excessively synchronized rhythmic activity appears to be related to Parkinsonism. As discussed previously, it is now becoming apparent that rhythms of different frequencies interact within the same signal. What of interactions between different signals at different sites? Could these interactions be important in tracking clinical state in PD?

Phase coherence in the beta band across contact pairs within the same electrode separated by a few mms also correlates with bradykinesia–rigidity and this phase coherence has been found to account for up to 25% of motor variability.^61^ What of locations that are much farther apart? In addition to the evidence that local oscillations may signify something important about the Parkinsonian state, consensus is growing that oscillations may play a physiological role in the functional connectivity/binding of different spatially segregated neuronal populations across much greater distances.^62^ Applying this to PD, we find that beta oscillations are coherent across bilateral STNs and cerebral cortices, suggesting the existence of a bilateral network controlling beta.^14,63^ Moreover, the interactions between these spatially segregated rhythms (as measured through coherence) are dynamically modulated by movement and dopamine with phase lag and lead being frequency dependent.^64–66^ With one exception (cortico-cortico coupling), these measures of widely separated multisite interaction (coherence), although dopamine and movement responsive, have yet to be shown directly to be correlated with behavioral characteristics such as bradykinesia–rigidity.^14^ It remains to be established whether characterizing interactions between widely distributed neuronal populations may give a more accurate representation of dynamic clinical phenotype, particularly given the consideration that recording from more than one site raises practical implications such as increased risk of hemorrhage and infection.

## Personal programming, dynamic optimization, and model-based control

The aforementioned studies have been concerned with seeking reliable neurophysiological biomarkers that represent relevant clinical states and are consistent across time and across patients. This system of attempting to definitively identify well-defined signals can be contrasted with an alternative approach in which one uses learning algorithms to investigate LFP signal space in individual patients that then extract complex, nonlinear multidimensional personalized signal characteristics from their LFP rather than using unitary biomarkers. Such approaches have been used elsewhere in brain–computer interfacing (BCI) and software for this is now available through open source sites such as BCILAB.^67^

A learning paradigm of this sort requires a training period in which signals are correlated with clinical features and thus requires quantitative information on clinical features in a manner that is quick, continuous, and reliable. Accelerometers can be used to give feedback information on limb tremor and joysticks or gyrosensors on bradykinesia.^68–70^ The real-time continuous measurement of rigidity is more challenging, and although quantification of rigidity has been achieved, these methods would need adaptation before implementation into a BCI.^71,72^ It would seem impractical to implant multiple bilateral devices to quantify motor impairment, but this approach could be realized by telemetrically downloading learned algorithms after training periods in a clinical laboratory.

A further potential approach is to move away from static biomarkers for feedback control and to design computerized model-based control systems. These forms of systems are now ubiquitous outside of the health technology field (e.g., GPS tracking and navigational systems, autopilot systems) but have yet to be fully implemented in medicine. The reason for the delay in uptake probably relates to previously insufficient computing power, inadequate neurophysiological models, and the nonlinearity in biological systems. All of these obstacles are becoming surmountable and PD is theoretically well placed to benefit from this type of system given its ongoing dynamic nature. The field of model-based control theory grew out of the work by Kalman in the 1960s in which a maximum-likelihood filter was used to track a system's state and calculate changes that were needed to the system's control signals in order to return it to a desired condition. With improvements in the implementation of these systems in nonlinear environments with developments such as the unscented Kalman filter (UKF), they are now being implemented in realistic models of neuronal behavior (e.g., Fitzhugh–Nagumo model). A substantial advantage of these systems is that they seek and utilize the best possible parameters for monitoring and control even if they do not represent a real biophysical value but are merely abstractions. In effect, the model (or control filter) is synchronized with the real system (basal ganglia) through continuous tracking and feedback. Additionally, the models allow one to monitor and record from just one site, such as the STN, and infer what the rest of the network is doing. Schiff has reviewed the history and development of model-based control theory and discussed its application to PD, and Feng *et al.* have attempted to implement it in a model of PD to improve DBS stimulation through an evolutionary optimization system for control of DBS stimulation parameters and separately through a nonlinear feedback model.^73–75^

## Proof of principle

**Figure 3:**
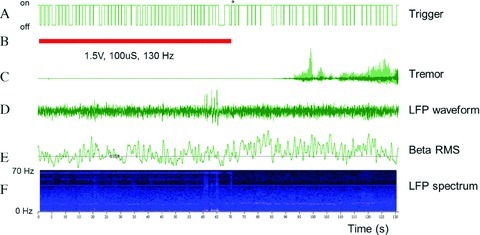
Closed-loop stimulation in a Parkinsonian patient with tremor. The bottom panel (F) shows the spectrogram of the LFP signal and demonstrates a low-frequency beta peak in yellow (original unfiltered LFP shown in D). The beta power in the form of beta root mean squared (RMS) is also displayed (E), along with the trigger threshold (horizontal line). Crossing of this threshold gave a positive trigger output that was sustained for a minimum of one second or until beta power dropped below threshold again. Panel (A) shows trigger output. Stimulation (1.5 mV, 100 μs, 130 Hz) was delivered while trigger output was positive during the first half of the recording, denoted by the red line (B). Trigger continued after this but did not result in stimulation. Accelerometer recording (C) demonstrates good tremor suppression during closed-loop mode (first half) with 26% reduction in stimulation triggering time. Note the increase in beta RMS during the second half of the recording when there is no stimulation. Previously unpublished data.

Whether it is possible, let alone feasible, to fully characterize the multifaceted nature of PD through examination of a single signal, such as the LFP, remains to be seen. Should this dissuade us from the goal of closed-loop therapy? Or can we still get quantitatively important improvement and superiority to standard open-loop DBS despite the richness of the Parkinsonian phenotype? A pioneering report from Bergman *et al*. suggests that this can be the case.^76^ They demonstrated that acute closed-loop stimulation was superior to standard DBS in monkeys when triggered, off, single-action potentials from individual neurons in the GPi or the M1 area of the cortex with an optimal delay of 80 ms. This exciting result provides proof of principle that closed-loop stimulation can be effective. Preliminary data from our group are also encouraging in suggesting that the differing pathophysiology of bradykinesia–rigidity versus tremor may not be so problematic, in so far as closed-loop control based on beta power levels in the LFP can, in practice, also control tremor (Fig. 3).

## The future

Feedback control of DBS has the potential to reduce power consumption/costs, improve efficacy, and reduce side effects. As the scope of DBS widens, feedback control will be crucial to fully realize the potential of this therapy.

In PD, the most fruitful feedback source for implementation into closed-loop DBS systems in the short-to-medium term is the LFP, given that it can be recorded from the stimulating electrode without further intervention and during stimulation. Much work has been directed toward the spectral analysis of the LFP recorded by the DBS electrode; here, the most consistent finding is a close relationship between beta activity and bradkykinesia–rigidity. However, this research has addressed pathophysiological questions and has not necessarily been tailored to assess whether signals might provide reliable feedback on current clinical state. Hence, the focus has been on correlations between subjects rather than within subjects (Table 3).

We highlight four priorities for future work in the field:

- More evidence has to be accrued for feedback signals that might correlate with Parkinsonian impairments other than bradykinesia–rigidity;
- Candidate signals for feedback control should be demonstrated to correlate with clinical state over the day and within the same subject, rather than across different subjects;
- The validity of biomarker signals, in particular the correlation between signal and clinical state, needs to be shown to be consistent over years within subjects; and
- The extent to which correlating brain signals are causally important needs to be established before stimulation can be specifically patterned, rather than delivered at empirically defined high frequency.

In the longer term, it is possible that a more detailed representation of clinical state for feedback control can be ascertained via more complex analyses, such as higher-order spectral analyses (bispectrum), phase–amplitude relationships, multisite recordings, and model-based control systems. However, a balance has to be struck between complexity of analysis versus processing time and power consumption, and the eventual choice of biomarker for feedback will depend on progress in both neuroscience and engineering/computer science. It is imperative that research continues apace to establish which of these markers holds the highest fidelity to the clinical state and to advance the implementation of these signals into a working responsive deep brain stimulation system. This will require cross collaboration between neurologists, neurosurgeons, engineers, computer scientists, and industry.^77,78^ It is clear that our present knowledge relating underlying signals to clinical state within patients as required for a closed-loop system is limited and requires extensive further research before this promising technology can be actualized.
